# Reexpansion pulmonary edema after drainage of tension pneumothorax

**DOI:** 10.11604/pamj.2015.22.143.8097

**Published:** 2015-10-15

**Authors:** Adriá Rosat, Carmen Díaz

**Affiliations:** 1 Department of General Surgery, Hospital Universitario Nuestra Señora de Candelaria, Ctra, Del Rosario 145, 38010 Sta, Cruz de Tenerife, Spain

**Keywords:** Pulmonary edema, drainage, tension pneumothorax

## Image in medicine

A 26-years-old man was admitted to our hospital emergency room for a traumatic pneumothorax four days after a fight. He had dyspnea, decreased blood pressure, tachycardia and tachypnea. Chest Xray showed a left pneumothorax with midline structures desviation and he was treated by intercostal drainage. 30 minutes later he started again with dyspnea and oxygen saturation decreased despite the addition of oxygen. A new chest Xray revealed a left reexpansion pulmonary edema. Glucocorticoids, diuretic stimulants, analgesic and bronchodilatators were administered in the intensive care unit. Gradually, the edema and dyspnea diminished and the patient could be discharged in good clinical condition. Reexpansion pulmonary edema (RPE) is a clinical syndrome characterized by the development of clinical and radiographic evidence of pulmonary edema, most commonly seen after large volume (>1.5 L) therapeutic thoracentesis. It is a rare complication (reported incidence approximately 0.8%) but is associated with a mortality rate up to 20%. Younger age, chronicity of the pneumothorax beyond three days, its large extends and a quick lung reexpansion have been identified as risk factors for the development of RPE. The pathogenesis of RPE is probably related to histological changes of the lung parenchyma and reperfusion-damage by free radicals leading to an increased vascular permeability. RPE is often self limiting and treatment is supportive.

**Figure 1:**
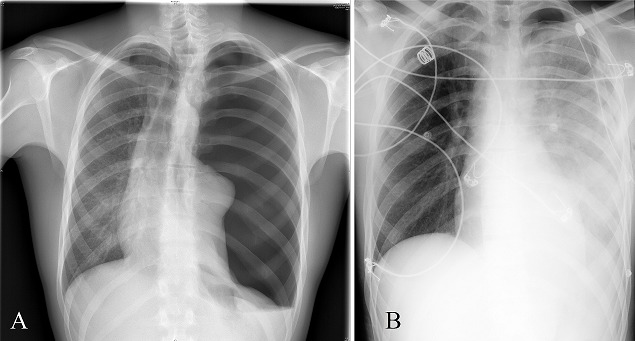
(A) left tension pneumothorax with midline structures desviation to the right; (B) postprocedure chest Xray showed near-immediate reexpansion of the left lung with new infiltrates in the whole lung

